# Relationship Between the Use of Fitness Trackers and Smartwatches for Monitoring Physical Activity and the Sociodemographic Characteristics of Long-Term Care Residents During the COVID-19 Lockdown

**DOI:** 10.3390/medicina61010006

**Published:** 2024-12-25

**Authors:** Ivana Crnković, Karmen Lončarek, Nada Tomasović Mrčela, Danica Železnik, Tomislav Vlahović

**Affiliations:** 1 Department of Physiotherapy, University of Applied Health Sciences, 10 000 Zagreb, Croatia; 2 Department of Ophthalmology, Faculty of Medicine, University of Rijeka, 51 000 Rijeka, Croatia; 3 Department of Public Health Gerontology, Andrija Štampar Teaching Institute of Public Health, 10 000 Zagreb, Croatia; 4 University Department of Health Studies, University of Split, 21 000 Split, Croatia; 5 Faculty of Health and Social Sciences Slovenj Gradec, 2380 Slovenj Gradec, Slovenia; 6 Clinic for Traumatology, Clinical Hospital Center Sestre Milosrdnice, 10 000 Zagreb, Croatia; 7 Department of Clinical Medicine, University of Applied Health Sciences, 10 000 Zagreb, Croatia

**Keywords:** COVID-19 lockdown, long-term care residents, activity trackers, physical activity

## Abstract

*Background and Objectives*: The use of wearable fitness technology is a trend nowadays and has significant potential in promoting an active lifestyle among long-term care (LTC) residents. The objectives of this observational study were to examine the use of fitness trackers and smartwatches for monitoring physical activity and to analyze the relationship between the use of these technological solutions and the sociodemographic characteristics of LTC residents during the COVID-19 lockdown. *Materials and Methods*: Face-to-face interviews were conducted with 198 LTC residents stationed in eleven organizational units that provide long-term accommodation services for older adults in the city of Zagreb in Croatia. LTC residents aged 65 and older who receive the 1st level of accommodation services in the social care system according to their functional ability and health status were included in this study. *Results*: During the COVID-19 lockdown, 19.19% of LTC residents used wearable activity trackers. Gender (*p* = 0.0411) and education level (*p* = 0.0485) were recognized as significant sociodemographic predictors regarding the use of fitness trackers and smartwatches for monitoring physical activity. An odds ratio for gender of 0.454 (95% CI: 0.213–0.969) indicates that women have a 54.6% lower chance of using fitness trackers and smartwatches then men. The odds ratio for the education effects of 0.050 (95% CI: 0.003–0.980) demonstrates that there is a 95% lower chance of using fitness trackers and smartwatches for individuals with only elementary education as opposed to university graduates. *Conclusions*: The sociodemographic differences of LTC residents regarding the use of fitness trackers and smartwatches require further research, but they are also an incentive for the implementation of these technological solutions to protect the health of older adults.

## 1. Introduction

The inclusion of an older adult in regular physical activity has a direct impact on their health and quality of life. The complex doctrine of regular physical activity in older age significantly contributes to the prevention of chronic non-communicable diseases, reducing the rate of bone loss, and improving the body’s immune response, as well as anthropometric characteristics, cognitive and functional abilities, and independence from the help of others [1,2]. It also directly affects the maintenance of mental health, which ranks it among the key issues of global public health policy [1,2]. Despite the recommendations of renowned world organizations, whose expertise supports the implementation of the recommended level of physical activity for all age groups, studies suggest worrying results that indicate that practicing physical inactivity is a significant cause of chronic non-communicable diseases and mortality at the global level [3,4,5]. Long-term institutional care (LTC) residents in the older age group are an extremely vulnerable group of society in which there are often behavioral changes in terms of reducing physical activity that negatively affect health outcomes [6]. Therefore, there is a great need for support introduced by a multidisciplinary gerontological team with an aim of providing geroprophylactic measures for maintaining and improving functional ability and prevention of disease through ensuring the recommended level of physical activity. The daily level of physical activity with an individual gerontological approach is adapted to the level of functional ability and the health status of the older adult [2,7].

The use of wrist-worn wearables in the older age group for the purpose of monitoring physical activity is no longer the future, but a reality that has significant potential in proactive geriatric care. Wearable technological solutions by their nature of objectivity avoid errors caused by incorrect interpretation, overestimation, and social desirability when assessing physical activity [8,9,10]. Such technological solutions contribute to the objective assessment of and increase in physical activity in the older age group [11], and scientific knowledge about this is provided by conducted studies which have incorporated wearable trackers for monitoring the activity of older adults in long-term care into their projects [12,13] and recorded positive results in the that domain [14,15]. Despite the positive and encouraging results on the significant contribution of wearable technological solutions in the dimension of physical activity among LTC residents, which has a significant contribution in the context of public health policy, the use of wearable trackers for monitoring activity in long-term care institutions is still insufficiently represented in the scientific literature. In addition to that, it was precisely the “new normal” era during the COVID-19 pandemic, which resulted in a significant reduction in physical activity and a noticeable motor and functional decline in the older age group [16,17], that opened up new research questions about heterogeneity when it comes to using wrist-worn wearables and sociodemographic characteristics of LTC residents which are important gerontological and public-health indicators for the design of individualized gerontological programs and a set of geroprophylactic measures [18].

Based on the above, the objectives of this observational study were to examine the use of fitness trackers and smartwatches for monitoring physical activity and to analyze the relationship between the use of these technological solutions and the sociodemographic characteristics of LTC residents during the COVID-19 lockdown period.

## 2. Materials and Methods

### 2.1. Study Design and Participants

This research was approved by the Social Protection and Disabled Persons’ Office of the City of Zagreb (CLASS: 550-01/21-001/241; Registration number: 251-17-22-1/3-21-2). Additionally, this research was approved by the Ethics Committee of the Faculty of Medicine of the University of Rijeka (CLASS: 003-08/19-01/93; Registration number: 2170-24-19-2). The study protocol ensured that the data collection by the respondents was in accordance with ethical and bioethical principles, which ensured voluntary participation in this research, confidentiality, security, privacy, and protection of the secrecy of the collected data in accordance with the Declaration of Helsinki.

This observational study initially included 227 long-term care residents stationed in eleven organizational units that provide long-term accommodation services for older adults under the jurisdiction of the City of Zagreb in Croatia [19]. This research was conducted in the post-lockdown period from May to October 2021. Before conducting this research, the managers of LTC institutions for older adults approved the arrival of the research team, which was obliged to comply with the Recommendation for the Preservation of the Health of Persons Aged 60+ and Persons Suffering From Chronic Diseases and Protection Measures Against Respiratory Infections (Including SARS-CoV-2) for Persons with Chronic Diseases and Older Adults and the Recommendation for the Preservation of the Health of Persons Over 65 Years of Age and the Chronically Ill (COVID-19) [20,21,22].

**Figure 1:**
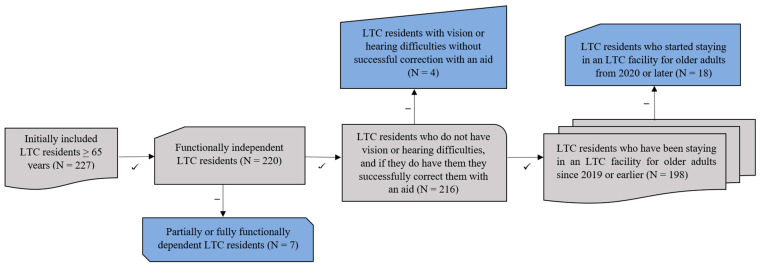
Concept of the selection of research participants.

Before the selection of participants, an informative meeting was organized where interested LTC residents were given detailed instructions about the study protocol by the researcher. Only the residents who signed an informed consent and are 65 years old or older, functionally independent individuals who independently meet their needs without the help or supervision from another person and who receive the 1st level of accommodation services in the social care system [19], do not have vision or hearing difficulties, and if they do have them they successfully correct them with an aid, and have been staying in an LTC facility for older adults since 2019 or earlier participated in this study. Residents which did not sign an informed consent, were under 65 years of age, partially or fully functionally dependent individuals who need help or supervision from another person in the 2nd–4th level of accommodation services in the social care system [19], had vision or hearing difficulties without successful correction with an aid, and started staying in an LTC facility for older adults from 2020 or later could not participate in this study. A total of 198 long-term care residents were included in the data analysis. The concept of the selection of research participants is shown in Figure 1.

### 2.2. Data Collection

The selected sociodemographic characteristics of participants were identified in 4 parts with closed questions through a face-to-face interview with the researcher. The questionnaire was created by a research team with expertise in the field of gerontological practice. Participants had to report their chronological age (years as a whole number), gender (women; men), level of education achieved (elementary, high school, and university) and marital status (single, married or living as married, divorced, and widowed). The use of fitness technological solutions such as fitness trackers and smartwatches for the purpose of monitoring physical activity during the COVID-19 lockdown period in 2020 according to the participants’ self-assessment was evaluated as a dichotomous variable (did not use/used). Participants who have used fitness trackers or smartwatches to monitor one or more indicators of physical activity such as the number of achieved steps, walking distance, physical activity energy expenditure or active minutes at least once a week were categorized as a group that was using wearable technological solutions for the purpose of monitoring physical activity. Participants who have monitored one or more indicators of physical activity such as the number of achieved steps, walking distance, physical activity energy expenditure, or active minutes using fitness trackers and smartwatches less than once a week or who have not used those devices at all were categorized as a group that was not using wearable technological solutions for the purpose of monitoring physical activity. During the interview, the researcher asked the participants whether the constructed questions were clear and understandable to them. The time needed to complete the questionnaire ranged from 5 to 7 min.

### 2.3. Data Analysis

Univariate descriptive statistics and bivariate analyses were used and presented in the initial part of this study. To examine the relationship between sociodemographic variables and the likelihood of using fitness trackers or smartwatches, univariate logistic regression models were conducted. In these models, the use of wearable trackers was treated as a binary outcome variable, with each of the four sociodemographic variables analyzed as potential predictors. In the multivariate logistic regression analysis, three multivariate models, incorporating sociodemographic variables as predictors, were evaluated, and the results of the selected model are presented. Statistical significance was defined as *p* < 0.05 (two-tailed). All analyses were performed using SAS© version 9.4 (Cary, NC, USA) [23].

## 3. Results

### 3.1. Descriptive Statistics

Table 1 shows the descriptive statistics for the numerical variable of participants’ age and the summary statistics for categorical sociodemographic variables (gender, education, and marital status). It could be observed that (for the 198 participants in the sample) the mean age was 81.15, with a standard deviation of 4.45. The youngest participant was 72, and the oldest was 102 years old. The median age was 81. According to the results in this study, a total of 73.2% of the participants included in this study declared themselves as women, while 26.77% of the participants in this study declared themselves as men. Most of the participants in this study have attained a high school level of education (73.74%), declare themselves as widows/widowers (59.60%), and live in a married or cohabiting union (22.22%). During the COVID-19 lockdown period, 19.19% of participants used fitness trackers and smartwatches to monitor their physical activity.

**Figure 2:**
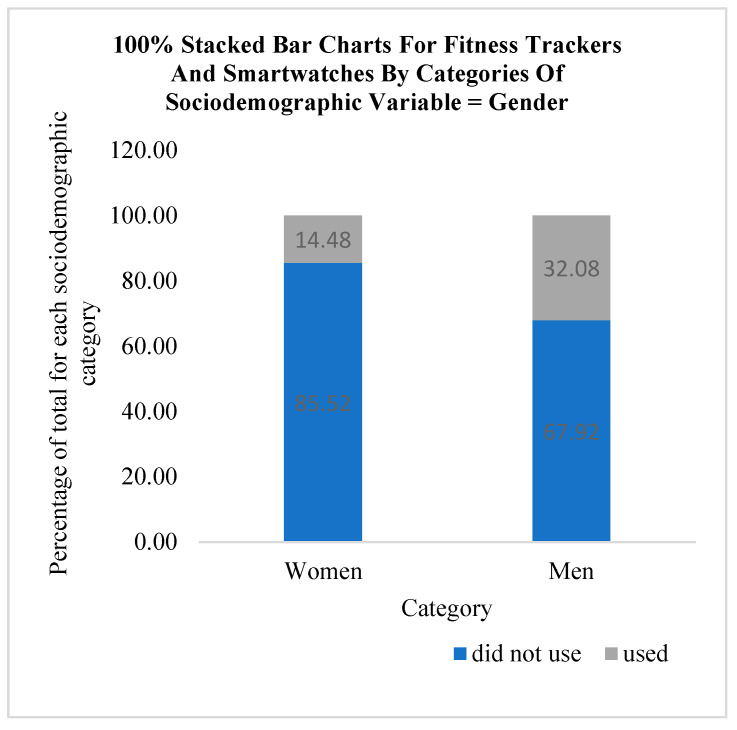
Using fitness trackers and smartwatches by categories of gender.

**Figure 3:**
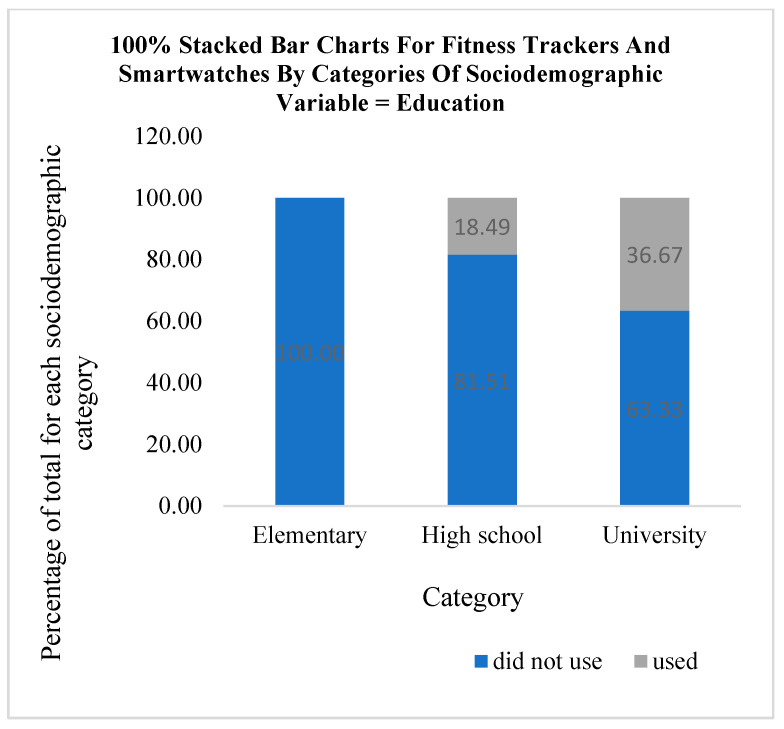
Using fitness trackers and smartwatches by categories of education.

**Figure 4:**
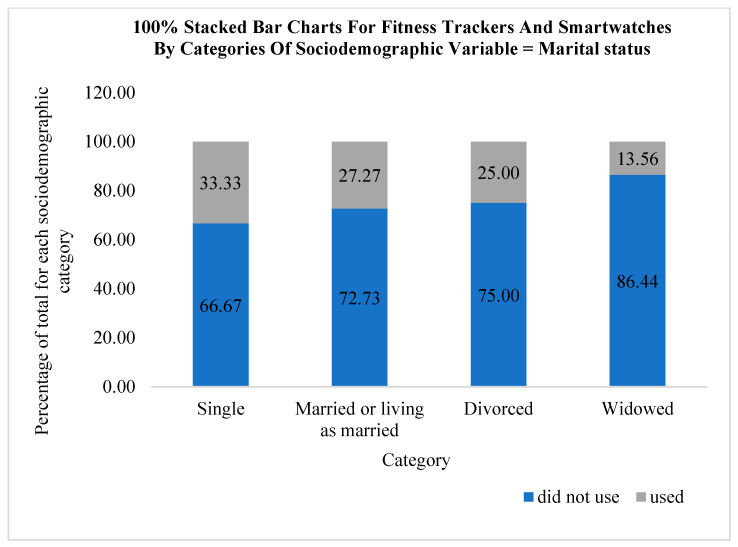
Using fitness trackers and smartwatches by categories of marital status.

The use of fitness trackers and smartwatches for monitoring physical activity according to categories of sociodemographic variables is graphically presented using stacked bar charts in Figure 2, Figure 3 and Figure 4. According to the results of this research, out of a total of 38 participants who used fitness trackers and smartwatches, a higher percentage of men (32.08%, N = 17) use these technological solutions compared to women (14.48%, N = 21). It can be observed that fitness trackers and smartwatches were used only by the participants with a university (36.67%, N = 11) or high school education (18.49%, N = 27). With regard to the category of marital status, it can be observed that the participants who declared themselves as single used these technological solutions the most (33.33%, N = 4) compared to the people living in a married or cohabiting union (27.27%, N = 12) and separated individuals (25.00%, N = 6). The people who declared themselves as widows/widowers used fitness trackers and smartwatches the least in the COVID-19 lockdown period (13.56%, N = 16).

### 3.2. Univariate Logistic Regression

The results in Table 2 indicate that there is a statistically significant relationship between the probability of using fitness trackers and smartwatches and gender (*p* = 0.0074) and education level (*p* = 0.0027), whereas no statistically significant relationship was found for either marital status (*p* = 0.1048) or age (*p* = 0.1383).

Univariate logistic regression results for gender (presented in Table 3) demonstrate that women have a 64% lower chance of using fitness trackers and smartwatches then the male participants.

Moreover, the odds ratio estimate for the education effects (of 0.39) demonstrates that there is a 61% lower chance of using fitness trackers and smartwatches for individuals with only a high school degree, as opposed to university graduates (as is displayed in Table 4). Likewise, individuals with elementary education have a 96% lower chance of using these technological solutions than those with a university degree.

Because of the quasi-complete separation of data points, the univariate logistic model for education with penalized likelihood method originally proposed by Firth [24] was used instead of the standard (Fisher scoring) unconditional likelihood method.

### 3.3. Model 3 Results

**Figure 5:**
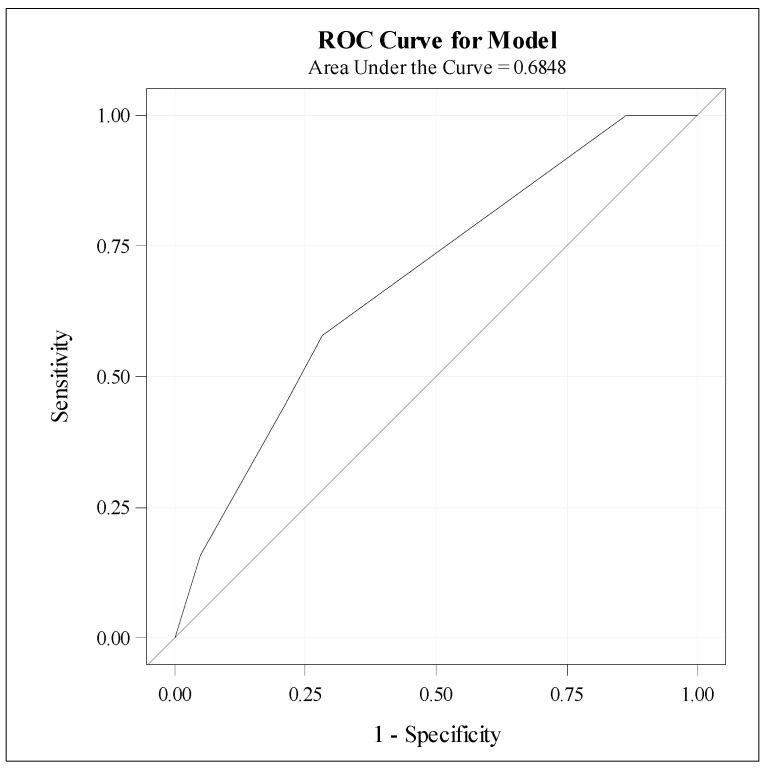
Roc curve for Model 3.

Although the AUC indicates moderate discriminative ability (Supplementary Materials), as further illustrated by the ROC curve (Figure 5), Model 3 is well suited for understanding the relationship between predictors and the outcome, given the constraints of the data.

Based on Penalized Maximum Likelihood Estimates, gender (*p* = 0.0411) and education-elementary vs. university (*p* = 0.0485) were significant, while education-high school vs. university was not a significant predictor (*p* = 0.0776) (Table 5).

**Figure 6:**
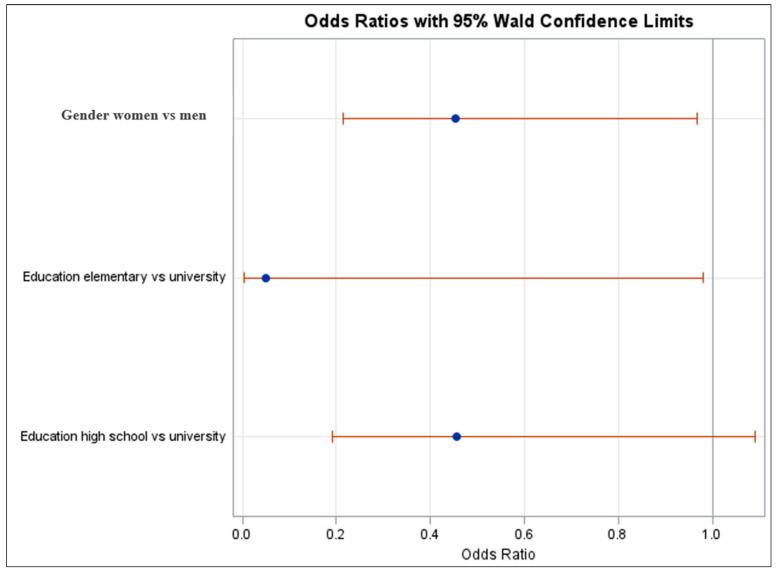
Odds ratio plot with 95% Wald confidence limits for Model 3.

An odds ratio for gender of 0.454 (95% CI: 0.213–0.969) indicates that women are 54.6% less likely to exhibit the outcome compared to men, holding other variables constant. Similarly, individuals with only elementary education are 95% less likely (odds ratio: 0.050; 95% CI: 0.003–0.980) to exhibit the outcome compared to those with a university education, holding other variables constant. However, the comparison between high school and university education was not statistically significant, as the confidence interval for the odds ratio of 0.456 (95% CI: 0.191–1.091) includes 1, indicating no conclusive difference between these groups (Figure 6).

**Figure 7:**
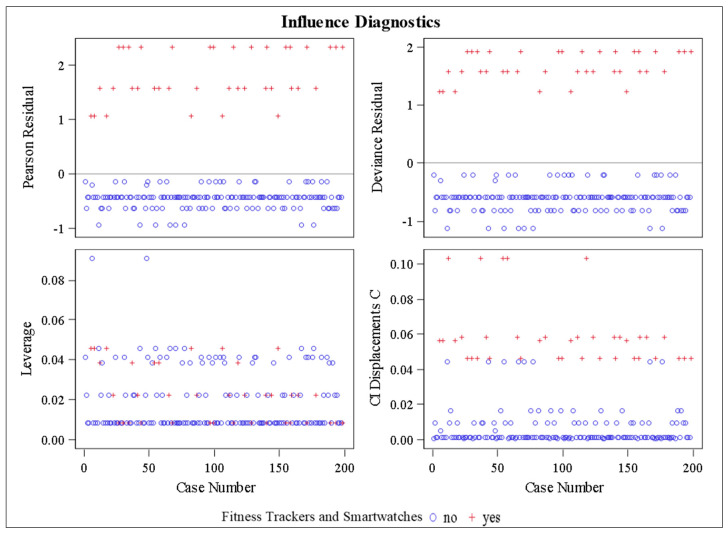
Influence diagnostics plot.

A few data points may be outliers or exert some influence, as indicated by Leverage and CI Displacements C. The absence of clear patterns in residual plots suggests no severe violations of model assumptions (Figure 7).

## 4. Discussion

Despite the attractiveness and numerous advantages provided by the fitness technology in this study, only 19.19% of LTC residents used fitness bracelets and smartwatches for the purpose of monitoring physical activity during the COVID-19 lockdown period. These results are in line with the existing research on older adults in the period before and during the COVID-19 pandemic. Schlomann et al. report that 18.9% of people aged 50 or older use mobile devices for tracking physical activity [25]. Similar results are reported by Seifert and his research team, where a total of 20.5% of older adults use mobile devices for tracking physical activity [26]. Jiang and his research team state that the prevalence of using wearable devices for tracking physical activity in the older age group, especially in people with known cardiovascular diseases or at risk, is still low, i.e., from 16% to 14%, but that the use of these technological solutions is an important modality in the dimension of physical activity [27]. In the elaboration of using fitness technological solutions in the older age group, it is necessary to consider the factors that influence the use of information and communication technology (ICT), which can contribute to health and well-being and the promotion of an active lifestyle. Studies indicate that the relevance and specificity of content aimed at the individual needs of the digital user, previous experience, and education and support from the environment, as well as technical specifications, are significant moderators that influence the use of ICT for the purpose of decision-making related to health in the older age group [28,29,30,31]. Even though the COVID-19 period contributed to the digital transformation of society and also to the greater inclusion of the older age group in the digital world, studies suggest a weak inclusion of ICT solutions that can contribute to the health and well-being of LTC residents, and the results of which also follow the COVID-19 period [32,33,34,35]. For LTC residents, even if they were motivated to use fitness bracelets and smartwatches in view of the implementation of anti-epidemic measures that resulted in the limitation of sports and recreational content in an LTC institution, having an ICT device that has the ability to monitor fitness activities is of crucial importance in the integration of this technology into their everyday life. Montgomery and his research team report on the use of ICT during the COVID-19 pandemic by nursing home residents and state that 40% of residents indicated that they had a device that enabled them to interact more easily on the web, 47% of residents indicated that the nursing home had computers or tablets, while 67% of residents indicated that the institution had provided them with unlimited access to the internet via Wi-Fi [36]. In the interpretation of the results of this study, it is necessary to consider how the willingness of LTC residents to use fitness technological solutions, in addition to the previously mentioned moderators, may have been influenced by the individual’s personal assessment of technical characteristics such as the dimensions of the device, practicality of the interface, and ease of use. In addition to the above, previous experience of LTC residents in the use of fitness technology, possession of digital knowledge and skills, support from members of the multidisciplinary gerontological team, as well as the support of family and friends, are of crucial importance and potentially influenced the residents’ interest for using these technologies during the COVID-19 lockdown period.

In our study, gender was recognized as a significant sociodemographic indicator for the use of fitness bracelets and smartwatches during the COVID-19 lockdown period, and the results suggest that women are 54.6% less likely to use this technological solution for monitoring physical activity compared to men. That gender is an important contextual factor in explaining the use of fitness technology before and during the COVID-19 pandemic is also suggested by other authors who conducted research in groups of older individuals. Regarding the use of fitness technology solutions, Seifert and his colleagues report that in the group of older adults, more men use smartwatches to monitor physical activity, but at the same time, they do not use activity trackers significantly more often than women [26]. The existence of differences between men and women in older age regarding the use of technological solutions and physical activity is also confirmed by the study conducted by Lee et al. [37]. According to research results, men are more encouraged to use smartphone applications to manage health if the applications can lead them into exercise routines. In explaining the difference between women and men in the use of wearable fitness technology, it is necessary to take into account the existence of digital inequality, which is reflected in access to the internet but also in the possibility of using modern technological solutions [38]. Access to information technology measured by owning a computer and computer literacy in the study by Kim et al. speaks of the maintained socioeconomic imbalance in the explanation of digital inequality in old age depending on the observed gender, where men accessed ICT solutions more often compared to women [39]. On the other hand, Bünning and his research team report that the time trend is a significant model that affects the use of ICT among men and women in middle and old age. In this study, in the period between 2014 and 2021, women equaled men in access to the internet and used it mostly for social contact. During the COVID-19 pandemic, Bünning et al. report that women have caught up with men in using the internet, especially in the context of entertainment content [40]. Therefore, the differences between women and men regarding the use of ICT solutions that have the possibility of monitoring fitness activities in LTC organizations during the COVID-19 lockdown are important to observe with regard to their personal interest and in the behavioral aspect. The implementation of current anti-epidemic measures, which resulted in the limitation of the involvement of family and friends in social contacts, possibly intensified the motivation in women to use ICT tools for the purpose of achieving communication, while men were more focused on the use of fitness technology.

Furthermore, in this study, the level of education achieved was recognized as a significant sociodemographic characteristic with regard to the use of fitness bracelets and smartwatches in the COVID-19 lockdown period. Compared to LTC residents with a university education, older individuals with only elementary education are 95% less likely to use wrist-worn wearables for monitoring physical activity. The results of available studies conducted before and during the COVID-19 period suggest that education among older adults is a significant sociodemographic indicator in the interpretation of using ICT for the purpose of practicing a healthy and active lifestyle [41,42,43,44]. According to the results of the research by Li L et al., a significant factor in the long-term use of wearable activity trackers among older adults is precisely higher education [41]. Morano and his research team report that in older age, a significant factor that correlates with the uptake of mHealth is an educational level higher than high school [42]. Jo HS et al. states that older adults with a higher level of education are more willing to use home-based healthcare ICT [43]. Md Fadzil NH and his research team report that in the COVID-19 period, among community-dwelling older adults with cognitive frailty, the use of digital technology was more common among individuals with a formal education of more than 6 years [44]. When elaborating differences in the use of fitness bracelets and smartwatches with regard to the level of education of LTC residents during the COVID-19 lockdown, it is necessary to use a multidimensional approach. Even though most ICT tools that have the ability to monitor fitness activities are available to the wider community due to their affordable price [45], the authors report on the cause-and-effect relationship between financial income and the use of ICT [27,43,46,47]. During the COVID-19 lockdown period, a potentially better socioeconomic status including a higher level of education was an important indicator, and it influenced the use of fitness technology among the LTC residents included in our study. Also, studies report a direct link between educational status and practicing physical activity in the older age group, which results in a significantly higher motivation to use fitness technological solutions [48,49]. It is possible that LTC residents with a higher level of education, despite the existence of movement restrictions due to the implementation of anti-epidemic measures, were more oriented towards involvement in physical activity, which directly affected their willingness to use wearable devices during the COVID-19 lockdown.

### 4.1. Practical Implications

Despite the fact that the fitness industry continuously follows trends aimed at practicing an active lifestyle among different age groups, additional investments by this sector in research and development of new technological solutions focused on the needs of the heterogeneous group of LTC residents are still necessary. The implementation of fitness technology in proactive geriatric care enables continuous monitoring with a tendency to sustain or improve the results achieved in the level of physical activity and fitness of the resident, maintaining and improving functional independence with the aim of integrating the individual into the local and wider social community. In addition to that, the integration of fitness technological solutions into the everyday life of LTC residents has an important potential in health promotion and prevention of chronic non-communicable diseases in the older age group. Also, additional educational workshops are needed by the multidisciplinary gerontological team on the protection of personal data when using these products, individual adaptation with regard to personal interests, health status and functional ability, daily and weekly schedules, as well as the possibilities of their application in the everyday life of LTC residents. Challenges in the application of fitness technological solutions for the purpose of gerontoprophylaxis and health promotion for older adults relate to additional adaptations according to their previously acquired preferences, such as the combination of traditional communication models with a leader and learning from books [50,51].

### 4.2. Study Limitations and Recommendations for Future Research

In interpretating the results of this study, it is necessary to take into account the existing limitations. It is necessary to take into account the possibility of user bias when answering and recall difficulties regarding the use of fitness technological solutions during the COVID-19 lockdown period. It is also desirable to conduct interventional and longitudinal studies in which it would be possible to investigate the connection between sociodemographic indicators of LTC residents and wearable devices, and the possibilities of their integration into modern proactive geriatric care. To examine the sociodemographic differences of LTC residents regarding the use of fitness technological solutions, in addition to age, gender, education, and marital status of the residents, it is necessary to include additional variables such as occupation, monthly income, presence of chronic diseases, use of tobacco products, and involvement in sports recreational activities. Additionally, our study used a relatively small sample of functionally independent residents in an urban environment. In new research, it is necessary to include a larger number of participants with different motor and functional abilities, with an additional check of the individual’s mental health status, which significantly affects the resident’s functional status, by a competent member of the multidisciplinary gerontological team [52]. It is also necessary to include LTC residents in non-urban areas and private institutions that provide long-term accommodation services for older adults in this new research.

## 5. Conclusions

During the COVID-19 lockdown, 19.19% of LTC residents used fitness trackers and smartwatches to monitor their physical activity. Gender (*p* = 0.0411) and education level (*p* = 0.0485) were recognized as significant sociodemographic indicators regarding the use of fitness trackers and smartwatches by LTC residents. Women are 54.6% less likely to use activity trackers compared to men. LTC residents with only elementary education are 95% less likely to use wearable technology for monitoring physical activity in contrast to university-educated residents. The results of this study contribute to the development of an individualized gerontological approach during the implementation of modern technological fitness solutions within integrated gerontological programs but are also an incentive for conducting a greater number of gerontological studies that have an aim of developing contemporary and proactive geriatric care.

## Figures and Tables

**Table 1:** Characteristics of participants included in this study.

Characteristic			
Age, Mean (SD)	81.15	4.45	
Gender, No. (%)	women	145	73.23
	men	53	26.77
Education, No. (%)	elementary	22	11.11
	high school	146	73.74
	university	30	15.15
Marital status, No. (%)	single	12	6.06
	married or living as married	44	22.22
	divorced	24	12.12
	widowed	118	59.60
The use of fitness trackers and smartwatches for monitoring physical activity in 2020, No. (%)	did not use	160	80.81
	used	38	19.19

**Table 2:** Univariate logistic regression results using each of the four sociodemographic predictors (likelihood ratio test results for the global null hypothesis β = 0).

Variable Name	Chi-Square	Pr > ChiSq
Age	2.1966	0.1383
Gender	7.1802	0.0074
Education	11.8066	0.0027
Marital status	6.1450	0.1048

**Table 3:** Odds ratio with 95% confidence interval for gender.

Odds Ratio Estimates and Profile-Likelihood Confidence Intervals				
Effect	Unit	Estimate	95% Confidence Limits	
Value women vs. men	1.0000	0.359	0.171	0.756

**Table 4:** Odds ratios and 95% confidence intervals for the education effects.

Odds Ratio Estimates and Profile-Likelihood Confidence Intervals				
Effect	Unit	Estimate	95% Confidence Limits	
Education elementary vs. university	1.0000	0.038	0.001	0.323
Education high school vs. university	1.0000	0.390	0.170	0.920

**Table 5:** Model 3 logistic regression results—analysis of Penalized Maximum Likelihood Estimates.

Analysis of Penalized Maximum Likelihood Estimates						
Parameter		DF	Estimate	Standard Error	Wald Chi-Square	Pr > ChiSq
Intercept		1	−0.5182	0.3848	1.8140	0.1780
Gender	women	1	−0.3945	0.1932	4.1719	0.0411
Education	elementary	1	−2.9901	1.5153	3.8938	0.0485
Education	high school	1	−0.7854	0.4451	3.1140	0.0776

## Data Availability

The data presented in this study are available upon request from the corresponding author.

## Supplementary Materials

The following supporting information can be downloaded at: https://www.mdpi.com/article/10.3390/medicina61010006/s1, Table S1. Wald Chi-Square values, degrees of freedom (DF) and the respective P-values for the effects in logistic models 1, 2 and 3. Table S2. Goodness of fit and Model Fit Statistics and the corresponding *p*-values (where available) for Models 1, 2 and 3.
