# Supplementary material for: Relationship Between the Use of Fitness Trackers and Smartwatches for Monitoring Physical Activity and the Sociodemographic Characteristics of Long-Term Care Residents During the COVID-19 Lockdown
Source: Medicina (Kaunas). 2024 Dec 25;61(1):6. doi: 10.3390/medicina61010006 (PMC11767113; doi:10.3390/medicina61010006)
Supplement: Supplementary file 1 [file medicina-61-00006-s001.zip › medicina-3344313-supplementary.pdf]

Model Selection and Comparison

Three multivariate logistic models were assessed and compared based on goodness-of-fit, model fit statistics (Table S2) and the significance of individual predictors (Table S1).

Model 1 included all four predictors: age, gender, marital status, and education. Model 2 included three categorical predictors: gender, marital status, and education, while Model 3 included only gender and education. Age was, as a predictor, included only in Model 1 due to its narrow span in a relatively small dataset (iqr=6). A limited range of age values can significantly reduce its effectiveness as a variable for explaining differences in the outcome.

Table S1. Wald Chi-Square values, degrees of freedom (DF) and the respective P-values for the effects in logistic models 1, 2 and 3.

	Model 1			Model 2		Model 3	
**Effect**	**DF**	**Wald Chi-Square**	**Pr > ChiSq**	**Wald Chi-Square**	**Pr > ChiSq**	**Wald Chi-Square**	**Pr > ChiSq**
**Age**	1	0.1139	0.7357				
**Gender**	1	2.2448	0.1341	2.3734	0.1234	4.1719	0.0411
**Marital_status**	1	1.2786	0.2582	1.2528	0.2630		
**Education**	2	4.9406	0.0846	4.8746	0.0874	5.6976	0.0579

Table S2. Goodness of fit and Model Fit Statistics and the corresponding P-values (where available) for Models 1, 2 and 3.

	Model 1		Model 2		Model 3	
**Goodness of Fit and Model Fit**	**Statistic/ DF**	**P-value**	**Statistic/ DF**	**P-value**	**Statistic/ DF**	**P-value**
**AIC**	170.079		174.181		176.621	
**SC**	189.808		190.622		189.774	
**-2 Log L**	158.079		164.181		168.621	
**Hosmer and Lemeshow test**	4.1340/7	0.7642	1.9885/5	0.8507	0.7089/4	0.9502
**R-Square**	0.0831		0.0831		0.0774	
**Max-rescaled R-Square**	0.1415		0.1385		0.1277	
**Deviance Value/DF**	68.8613/77	0.7344	11.6215/6	0.0710	1.1897/2	0.5516
**Pearson Value/DF**	53.5984/77	0.9805	9.8365/6	0.1317	0.7089/2	0.7015
**AUC**	0.704		0.710		0.685	

The goodness-of-fit tests applied (Hosmer and Lemeshow, Deviance, and Pearson Chi-square tests) did not yield a P-value below 0.05 for any model, indicating no detectable lack of fit.

However, the ratio of Pearson and Deviance values to the number of degrees of freedom (DF) being greater than 1 for Model 2, excluded it from further consideration.

When comparing the other two models:

- Model 1 had the lowest value of AIC (170), the highest R-square (0.08) and Max-rescaled R-square (0.14). However, none of its predictors were statistically significant at the 0.05 level, suggesting potential multicollinearity among the predictors (apart from a relatively small sample size).
- Model 3, with fewer predictors, showed moderate fit but with somewhat weaker model fit statistics compared to Model 1 (AUC 0.685 vs. 0.704 and Max-rescaled R-Square 0.1277 vs. 01415). However, Model 3 showed comparable (slightly lower) value of SC statistics (189.774) to that of Model 1 (189.808).

Given these results, while Model 1 initially appeared preferable based on fit statistics, the lack of significant predictors undermines its utility.

As a result, Model 3, with its simpler structure, was chosen for further analysis. To address issues of quasi-complete separation in the data, the Firth method was applied to ensure more reliable parameter estimation.
